# Development of a theory-based self-management digital intervention to promote physical activity in people living with chronic obstructive pulmonary disease: Respir’air BPCO

**DOI:** 10.1016/j.ijnsa.2026.100623

**Published:** 2026-07-11

**Authors:** Ricardo Salgado, Patrício Costa, Philippe Delmas, José Miguel Padilha

**Affiliations:** a La Source School of Nursing, HES-SO University of Applied Sciences and Arts Western Switzerland, Lausanne, Switzerland; b ICBAS - Abel Salazar Biomedical Sciences Institute, Porto University, Porto, Portugal; c RISE-Health, Nursing School of Porto (ESEP), Porto, Portugal; d Bureau d’Echanges des Savoirs pour des praTiques Exemplaires de Soins (BEST), Lausanne, Switzerland; e Center for Psychology at University of Porto, Faculty of Psychology and Education Sciences, University of Porto, Porto, Portugal; f Nursing School, University of Porto, Porto, Portugal

**Keywords:** COPD, eHealth, Intervention development, MRC, Nursing, Self-management

## Abstract

**Aim:**

To describe the development of Respir’air BPCO, a theory-driven self-management digital intervention to promote physical activity in people living with chronic obstructive pulmonary disease (COPD) following pulmonary rehabilitation.

**Background:**

Physical activity is a key component of COPD self-management, yet activity levels often decline after discharge from pulmonary rehabilitation. Digital interventions may extend support beyond face-to-face care, but existing interventions are heterogeneous and frequently lack explicit theoretical grounding. Involving patients and healthcare professionals in intervention development is essential to enhance acceptability and clinical relevance.

**Method:**

Development was guided by the updated Medical Research Council framework for complex interventions and operationalized using pragmatic elements from Bleijenberg’s approach. The process included evidence synthesis (umbrella review), theory selection (Middle-Range Theory of Self-Care of Chronic Illness and Self-Determination Theory), stakeholder needs assessment through focus groups with people living with COPD and healthcare professionals, contextual assessment in a Swiss pulmonary rehabilitation center, and modelling of processes and outcomes through a logic model. Content validity was assessed through a two-round modified e-Delphi with an expert panel.

**Results:**

Respir’air BPCO is a mobile application-based intervention comprising: (1) eight structured educational modules targeting physical activity, (2) daily step monitoring and personalized goal setting, and (3) motivational features including reminders and feedback. The intervention components were designed mainly to strengthen knowledge, self-management competencies, and motivation. Respir’air BPCO is expected to be feasible and acceptable, and to promote physical activity, improve disease self-management and motivation, enhance health-related quality of life, reduce symptom severity, and decrease exacerbations and hospitalizations.

**Conclusion:**

Respir’air BPCO was developed through a structured, theory-based, and stakeholder-informed design process involving patients and health professionals. Feasibility and acceptability will be evaluated in future studies.

**Practice implications:**

The intervention may help extend self-management support after rehabilitation and promote physical activity maintenance in daily life of COPD patients.

**°**

**What is already known**

• Physical activity is a core component of COPD self-management; however, activity levels commonly decline after discharge from pulmonary rehabilitation.
• Self-management digital interventions for people living with COPD can enhance access to supportive resources and may improve physical activity and quality of life, but existing interventions are highly heterogeneous, insufficiently described, and frequently lack explicit theoretical grounding.

**What this paper adds**

• This paper describes a systematically developed, theory-based self-management digital intervention, guided by the Medical Research Council framework, to promote physical activity in people living with COPD after pulmonary rehabilitation.
• The intervention integrates the Middle-Range Theory of Self-Care of Chronic Illness, Self-Determination Theory, and behavior change techniques, together with patient and healthcare professional input and evidence from the literature, to target key mechanisms underlying sustained physical activity behavior.
• This paper provides a transparent and reproducible model for developing clinically relevant complex self-management digital interventions for people living with COPD.

Alt-text: Unlabelled box dummy alt text

## 1 Background

Chronic obstructive pulmonary disease (COPD) is a major global health concern and is currently ranked among the leading causes of death worldwide (Global Initiative for Chronic Obstructive Lung Disease, 2026; WHO, 2024). It affects hundreds of millions of people globally, including around 400,000 in Switzerland (Adeloye et al., 2022; Global Initiative for Chronic Obstructive Lung Disease, 2026; OFSP, 2018). COPD is characterized by persistent airflow limitation and progressive respiratory impairment, largely driven by long-term exposure to tobacco smoke and environmental or occupational pollutants (Global Initiative for Chronic Obstructive Lung Disease, 2026). Beyond respiratory symptoms, COPD is associated with significant functional limitations, psychosocial burden, and reduced quality of life for individuals and families (Johansson et al., 2019). The disease also generates considerable healthcare costs, and its impact is expected to increase further due to population ageing and workforce shortages in healthcare (Boers et al., 2025; Global Initiative for Chronic Obstructive Lung Disease, 2026; Page et al., 2023).

Given the chronic and progressive nature of COPD, maintaining functional capacity and preventing avoidable deterioration are therefore key priorities in COPD care, particularly within secondary and tertiary prevention (Global Initiative for Chronic Obstructive Lung Disease, 2026; Holland et al., 2021; Schrijver et al., 2022). Effective self-management is widely recognized as a cornerstone of COPD care (Global Initiative for Chronic Obstructive Lung Disease, 2026), as it supports individuals—together with families, communities, and healthcare professionals—in managing treatments, adapting lifestyle behaviors, and addressing the broader consequences of living with a chronic condition (Matarese et al., 2018; Padilha, Sousa and Pereira, 2018; Richard and Shea, 2011; Riegel, Jaarsma and Strömberg, 2026). Nurses and allied health professionals play a central role in supporting these processes, informed by their close patient relationships, clinical expertise, professional competencies, and disciplinary knowledge. Structured self-management interventions, often incorporating behavior change techniques, aim to strengthen motivation, confidence, and long-term health behavior adoption (Effing et al., 2016; Pinto et al., 2025). In COPD, such approaches have been associated with improved quality of life and reduced healthcare utilization (Lenferink et al., 2017; Schrijver et al., 2022).

Physical activity is a central component of COPD self-management because it contributes to preserving function and limiting deconditioning (Global Initiative for Chronic Obstructive Lung Disease, 2026). It is defined as any bodily movement produced by skeletal muscles that induces energy expenditure (Caspersen, Powell and Christenson, 1985; WHO, 2022). Examples include domestic, leisure, and professional activities are examples of physical activity (Caspersen, Powell and Christenson, 1985). Sustained engagement in physical activity has been associated with improved quality of life and reduced risk of exacerbations, hospitalizations, and mortality (Global Initiative for Chronic Obstructive Lung Disease, 2026; Vogelmeier et al., 2017; Xiang et al., 2022). Physical activity promotion is commonly addressed within pulmonary rehabilitation (Global Initiative for Chronic Obstructive Lung Disease, 2026; Holland, Corso and Spruit, 2021). However, access to pulmonary rehabilitation remains limited, and completion rates are suboptimal (S. E. Desveaux et al., 2015; Global Initiative for Chronic Obstructive Lung Disease, 2026; Holland, Corso and Spruit, 2021; Jones et al., 2014b; Levack et al., 2018; Silva et al., 2024; Spitzer et al., 2019). Importantly, following discharge from pulmonary rehabilitation, many individuals lack structured support to sustain behavior change, leading to declining physical activity, progressive deconditioning, and a downward health trajectory characterized by increased morbidity and mortality, as well as poorer quality of life (S. E. Desveaux et al., 2015; Global Initiative for Chronic Obstructive Lung Disease, 2026; Holland, Corso and Spruit, 2021; Jones et al., 2014b; Levack et al., 2018; Watz et al., 2014).

Digital health interventions have increasingly been proposed as strategies to extend support beyond face-to-face care by improving accessibility, continuity of care, and the personalization of management and self-management support (A. Holland, Corso and Spruit, 2021; Jones et al., 2014a; Lear et al., 2021; Pinnock, Poberezhets and Drummond, 2023). These interventions may include mobile applications, wearable devices, telemonitoring systems, and videoconferencing technologies (WHO, 2016, 2023) and can support self-management interventions by providing remote support, facilitating real-time feedback, and promoting adherence to treatment plans (Effing et al., 2016; WHO, 2016). Evidence suggests that self-management digital interventions may reduce hospital length of stay and hospital admissions, decrease the number of exacerbations and levels of anxiety and depression, improve quality of life, enhance lung function, increase treatment adherence, and promote higher levels of physical activity (Janjua et al., 2021; Koh et al., 2023; Liu et al., 2023; Salgado et al., 2026; Yang et al., 2018). However, the current evidence base remains heterogeneous, with substantial variability in intervention components and insufficient reporting of key aspects such as the development process, intervention intensity, theoretical foundations, and outcome measures (Holland, Corso and Spruit, 2021; Janjua et al., 2021; Koh et al., 2023; McCabe, McCann and Brady, 2017; Salgado et al., 2026).

These limitations highlight the need for more clearly specified, theory-driven interventions that can be delivered consistently in clinical practice, including through the support of nurses and allied health professionals, who play a central role in promoting self-management and sustaining behavior change in people living with COPD.

## 2 Objective

The aim of this paper is to describe the development of Respir’air BPCO, a complex self-management digital intervention designed to promote physical activity in people living with COPD, informed by evidence, theory, and patients' and healthcare professionals' perspectives, validated by an expert panel and guided by the Medical Research Council (MRC) framework for complex interventions (Skivington et al., 2021).

### 2.1 Overall methods

The development of the intervention was guided by the updated Medical Research Council (MRC) framework for complex interventions. This framework conceptualizes intervention research as an iterative process comprising four overarching phases: development, feasibility and piloting, evaluation, and implementation. The present paper focuses exclusively on the development phase.

To maximize clinical relevance and ensure responsiveness to real-world conditions, the MRC framework was operationalized using a pragmatic approach informed by Bleijenberg et al. (Bleijenberg et al., 2018). Accordingly, particular emphasis was placed on: (1) identifying and defining the problem; (2) establishing the evidence base; (3) identifying or developing an underpinning theory; (4) assessing the needs of key stakeholders; (5) examining current clinical practice; (6) modelling intervention processes and outcomes; and (7) designing the intervention. In addition, a final step (8) was included to evaluate the intervention's content validity, further strengthening alignment among theoretical assumptions, stakeholder priorities, and practical feasibility.

To enhance transparency, reproducibility, and clinical usability, the description of the intervention followed the Template for Intervention Description and Replication (TIDieR) (Hoffmann et al., 2014) (Additional file 1). In addition, the Guidance for Reporting Intervention Development Studies in Health Research (GUIDED) framework was applied to support transparent documentation of the development process. (Duncan et al., 2020) (Additional file 2)

### 2.2 Target population

To maximize acceptability and clinical relevance, a target population–centered approach was adopted throughout the intervention's development. The self-management digital intervention was designed to promote physical activity in people living with COPD after completing a pulmonary rehabilitation program at a Swiss pulmonary center.

Given the complexity of COPD and the central role of self-management in disease outcomes, interventions for this population must align with patients’ capabilities, preferences, and lived experiences while remaining consistent with clinical recommendations. The present intervention was therefore developed to be autonomy-supportive, progressive, and accessible, enabling individuals to strengthen their self-management skills and integrate physical activity into daily life at their own pace.

### 2.3 Setting

The intervention was designed in partnership with a pulmonary rehabilitation center located in the French-speaking part of Switzerland. Individuals with COPD are admitted to a three-week inpatient pulmonary rehabilitation program, meaning they stay in the hospital full-time during the rehabilitation period, typically following a planned referral or an acute exacerbation. The center provides care for approximately 900 patients with COPD each year.

The rehabilitation program follows a structured 21-day clinical pathway that combines standardized components with an individualized exercise prescription based on baseline assessments. Patients participate in 21 supervised functional training sessions, delivered twice daily throughout the three-week hospital stay. These sessions integrate multiple components of pulmonary rehabilitation, including aerobic endurance training (e.g., cycling, treadmill walking, rowing, or Nordic walking), muscle-strengthening exercises using resistance equipment (machines, free weights, elastic bands, or body weight), breathing control techniques (e.g., diaphragmatic breathing, pursed-lip breathing, airway clearance strategies), and mobility and flexibility exercises delivered through adapted physical activity sessions. In addition, patients participate in six educational workshops covering key topics such as disease knowledge, smoking cessation, stress management, pharmacological treatment, physical activity, and nutrition.

After discharge, follow-up care is mainly delivered through outpatient consultations with pulmonologists and physiotherapy sessions, with limited opportunities for sustained behavioral support. This setting, therefore, provides a clinically relevant context for developing a digital intervention to extend self-management support beyond the rehabilitation period into patients’ everyday lives once they are back home.

## 3 Overview of the intervention development process

**Fig. 1:**
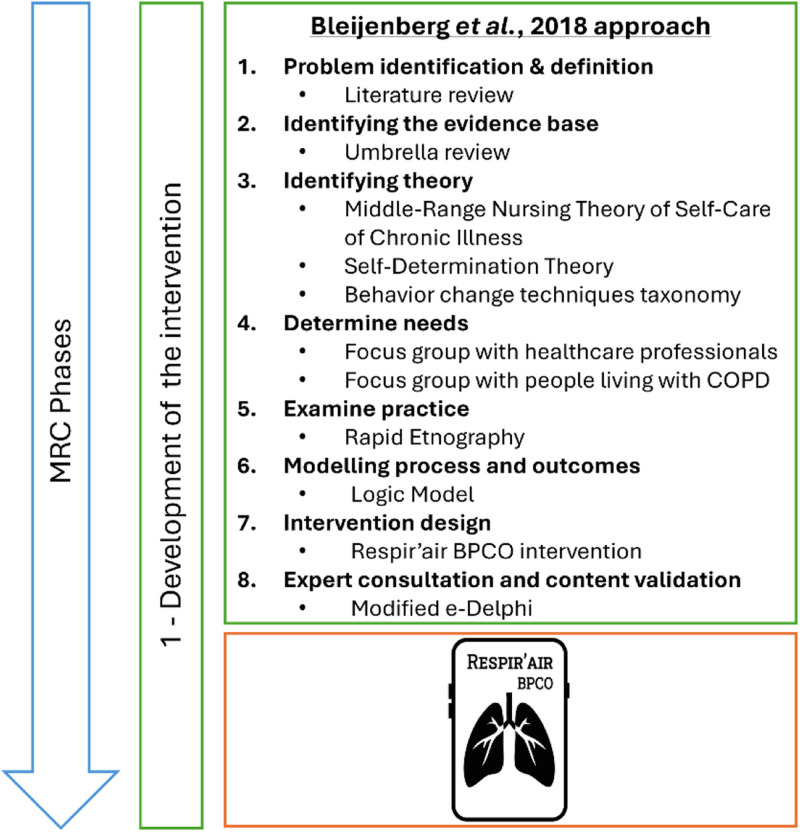
Intervention development overview.

Fig. 1 presents an overview of the development process and illustrates how these stages informed the final intervention since 2023.

### 3.1 Problem identification & definition

The problem was initially identified through empirical observations in clinical practice and was subsequently operationalized through a targeted literature review. This review aimed to develop an in-depth understanding of the challenges associated with sustaining physical activity as a core component of effective COPD self-management and to clarify the key concepts underpinning the intervention. Specifically, the review synthesized contemporary definitions, the most recent clinical recommendations, and conceptual boundaries related to self-management, physical activity in COPD, and self-management digital interventions. Establishing this conceptual foundation enabled a clear description of the current situation internationally and in Swiss territory, the desired post-rehabilitation program support, and the gaps in existing care and in the literature, as outlined in the Introduction.

### 3.2 Identifying the evidence base

Given the growing body of research on self-management digital interventions in COPD, several systematic reviews have been published in recent years. Therefore, to provide a comprehensive overview and up-to-date synthesis of the evidence base, an umbrella review was conducted to examine the effectiveness of self-management digital interventions on health-related outcomes (Salgado et al., 2025, 2026). The umbrella review synthesized the evidence of 13 systematic reviews and identified consistent evidence suggesting that self-management digital interventions may improve physical activity, health-related quality of life, lung function, less frequent exacerbations anxiety, depression, treatment adherence, knowledge acquisition, patient activation and self-efficacy. However, evidence regarding dyspnea, physical capacity, time spent in sedentary behavior, emergency visits, and mortality remains inconclusive (Salgado et al., 2026). Overall certainty of the evidence ranged from very low to moderate, and substantial heterogeneity was observed across intervention components, outcome measures, and study designs. Importantly, many interventions lacked an explicit theoretical underpinning to guide their development and evaluation (Salgado et al., 2026).

Together, these findings highlight the promising potential of self-management digital interventions for people living with COPD, while reinforcing the need for a theory-driven and systematically developed intervention, designed and reported in accordance with contemporary guidance for complex interventions.

### 3.3 Identify theory

Living with a chronic non-communicable disease such as COPD involves navigating multiple life transitions, defined as periods of instability between two states of relative stability and requiring psychological adaptation to change and disruption (Murphy, 1990). Following diagnosis and throughout the disease trajectory, individuals may experience health–illness, situational, and developmental transitions that can challenge their sense of balance and increase vulnerability (Meleis et al., 2000). In this context, nurses, drawing on their disciplinary knowledge (Fawcett and Desanto-Madeya, 2012) and clinical competencies, play a key role within interprofessional teams in supporting patients and families through these transitions by strengthening knowledge acquisition and understanding, reinforcing competencies and capacities, and empowering patients’ autonomy, thereby promoting effective self-management.

Given the complexity of physical activity behavior in COPD, and its central role in effective self-management, complementary theoretical perspectives were used to inform the intervention design and its mechanisms of action. The Middle-Range Nursing Theory of Self-Care of Chronic Illness provided the overarching framework, conceptualizing self-management through three core processes: maintenance (in which physical activity represents one of the key components), monitoring, and management (Riegel, Jaarsma and Strömberg, 2026). The theory further emphasizes that decision-making and individual reflection are inherent to self-management, and that optimal self-management is intentional, reflective, sufficient, and evidence-informed (Riegel, Jaarsma and Strömberg, 2026). In addition, it considers that self-management is influenced by individual and contextual factors, including experience and competence, motivation, culture, confidence, habits, functional capacity, symptom burden, social support, and access to care (Riegel, Jaarsma and Strömberg, 2026). Strengthening self-management abilities while using this theoretical framework in COPD has been associated with improvements in functional capacity, dyspnea management, and quality of life (Pinto, 2025). This theoretical perspective guided the identification of the core self-management processes to be targeted by the intervention and contributed to the selection and structuring of the educational content.

To better understand and address the motivational mechanisms underlying behavior change, Self-Determination Theory was used to guide the selection of intervention strategies to support motivation. According to Self-Determination Theory, individuals are more likely to initiate and maintain health behaviors when the basic psychological needs for autonomy, competence, and relatedness are supported (Ryan and Deci, 2017). These needs are particularly relevant in COPD, where individuals often experience reduced motivation due to symptom burden, fear of dyspnea, and reduced confidence in their physical capabilities (Xiang et al., 2022). Evidence suggests that interventions informed by Self-Determination Theory can enhance engagement in physical activity, support long-term behavior change, and improve both physical and emotional quality of life in people living with chronic conditions (Benzo et al., 2025; Teixeira et al., 2012). Therefore, particular attention was given to supporting the psychological needs for autonomy, competence, and relatedness throughout the intervention design (Ryan and Deci, 2000). To operationalize these principles, Self-Determination theory-consistent motivation and behavior change techniques taxonomy were selected using the expert consensus classification proposed by Teixeira et al. (Teixeira et al., 2019).

### 3.4 Determine needs

To complement the literature and contextual analysis, two focus groups were conducted to inform the intervention's content and design (Additional file 3). The first focus group (June 2024) involved six healthcare professionals (two nurses, two pulmonologists, and two physiotherapists) and explored perceived patient needs, barriers to physical activity, and feasible support strategies. Healthcare professionals highlighted learning needs in understanding COPD, symptom management (particularly dyspnea and anxiety), and physical activity recommendations and examples. Barriers to sustained physical activity were described as multifactorial, including symptom burden, fatigue, dyspnea, low motivation, limited social support, low health literacy, and contextual constraints such as oxygen therapy logistics, transport and infrastructure limitations, as well as limited post-discharge structured support framework. Participants highlighted the importance of simple, tailored content supported by behavior change strategies such as progressive goal setting, addressing misconceptions, positive reinforcement, problem-solving strategies, reminders, and opportunities for peer support.

The second focus group (July 2024) included six people living with COPD and explored needs and expectations regarding a self-management digital intervention promoting physical activity. Participants prioritized content on physical activity, exacerbation management, medication information, action planning, breathing control, energy conservation, motivation, stress and anxiety management, and clear information on whom to contact when support is needed. Desired features included a secure, user-friendly design and accessible formats with limited text, clear explanations, and greater use of visuals and videos; reminders; encouraging and non-judgmental feedback; motivational support; guidance on physical activity and progression; optional peer support with appropriate moderation; and the possibility of selecting a “rest day” option during more difficult periods.

Overall, the focus groups findings provided actionable insights that directly informed the intervention’s content, structure, design and delivery format. Key implications included the prioritization of core educational modules on self-management, physical activity, symptom management, the integration of behavior change strategies such as progressive goal setting, positive feedback, and reminders; and the inclusion of features designed to enhance engagement and acceptability, such as clear and concise content, visual support, and adaptable activity progression.

### 3.5 Examine practice

To ensure the intervention was grounded in real-world clinical practice, a context assessment was conducted using an immersive observational approach informed by rapid ethnography. Several days of immersion were undertaken within the partner Swiss pulmonary rehabilitation center, allowing observation of the care pathway from admission to discharge. This included patients’ interactions with key healthcare professionals (e.g., nurses, physiotherapists, physicians), as well as routine clinical activities, self-management support practices, pulmonary rehabilitation practices, transitions between care components, and organizational workflows.

These observations provided practical insight into everyday routines, contextual constraints, and opportunities to integrate a digital intervention after discharge. The aim was not to generate ethnographic theory, but to inform pragmatic design decisions and enhance alignment between intervention components and the implementation context. Importantly, this assessment highlighted that although patients receive intensive support during inpatient rehabilitation, they often lack readily available resources after discharge, which may compromise the maintenance of rehabilitation gains.

### 3.6 Modelling process and outcomes

A logic model was developed to clarify how intervention components were expected to influence outcomes (Additional file 4). It also illustrates in detail how the intervention is grounded in the Middle-Range Theory of Self-Care of Chronic Illness and Self-Determination Theory. Behavior change techniques were embedded across both the educational modules and digital features (education, reflection, goal setting, self-monitoring, and feedback). These components were designed to strengthen self-management competencies, autonomous motivation, and self-regulatory skills, thereby supporting sustained engagement in physical activity. Clinician stakeholder feedback confirmed the intervention’s clinical relevance and highlighted the importance of flexibility, personalization, and minimal burden to facilitate integration into routine care.

### 3.7 Intervention design

The final intervention, Respir’air BPCO, is a theory-based self-management digital intervention designed to promote physical activity in people living with COPD following completion of pulmonary rehabilitation. It was developed as a complementary solution providing accessible, asynchronous, structured, and patient-centered support within patients’ everyday environments.

The intervention is delivered through a mobile application built on the Breathment® mobile app (Android and iOS), which provides the technical infrastructure for the intervention content, and comprises three interrelated components (Table 1):

**Table 1:** Overview of the components, modules/features, and content of the Respir’air BPCO self-management digital intervention.

Components	Modules/Features	Content
A) Structured educational self-management modules focusing on physical activity	Introduction	Introduction to the mobile application and modules
	COPD	Definition of COPD
		COPD risk factors
		Signs and symptoms of COPD
		Diagnosis and stages of COPD
	Self-management	Definition of self-management
		Importance of effective self-management
		Key components of self-management
	Physical activity	Definition of physical activity
		Difference between physical activity and physical exercise
		Examples of physical activities
		Importance of physical activity
		Practical tips for physical activity
		Breathing techniques during physical activity
		Assessment of effort level
		Identifying limits
		Useful positions in case of dyspnea
	Getting started	Personal reflection on the pros and cons of physical activity
		Formulating progression goals (SMART approach)
		Identifying facilitators and barriers to physical activity
		Strategies for overcoming barriers to physical activity
		IF/THEN method for adapting to difficulties and unforeseen events
	Progression strategies	Step count progression strategy
	Energy conservation tips	Energy conservation strategies
	Action plan	Developing an action plan in case of deterioration/worsening
B) Physical activity monitoring and progression support	Step counter	Daily step count monitoring
	Activity history	Visualization of daily step history
	Goal setting	Personalized daily step goals
C) Motivational and supportive features	Motivational messages	Encouraging and supportive feedback messages
	Reminders	Automated reminders to support engagement
	Air quality information	Real-time outdoor air quality information based on user location

#### 3.7.1 A) Structured educational self-management modules

The core component of Respir’air BPCO consists of eight educational modules comprising 25 brief learning units, which users can consult at their own pace and in the order that best suits their needs. Modules are delivered using a combination of text, images, and short videos, designed to accommodate different learning preferences and levels of health literacy. The modules address the following themes:

##### 3.7.1.1 Introduction to the digital intervention

This module introduces the application, its objectives, and how it can promote physical activity and self-management. Users are encouraged to progress through the content at their own rhythm and reassured that all materials remain accessible at any time.

##### 3.7.1.2 COPD

Content provides information on COPD, including its definition, risk factors, signs and symptoms, diagnostic process, and disease stages. The aim is to enhance understanding of illness as a foundation for effective self-management.

##### 3.7.1.3 Self-management

This module presents self-management as a central component of living with COPD. It covers key elements such as symptom monitoring, medication management, avoidance of respiratory irritants, communication with healthcare professionals, emotional wellbeing, and social support, situating physical activity within a broader self-care framework.

##### 3.7.1.4 Physical activity

The content focuses on the role of physical activity in the management of COPD. It clarifies the distinction between physical activity and exercise, provides examples of everyday activities, and offers practical guidance on engaging in physical activity safely. Key content includes recognizing normal versus warning symptoms, using breathing techniques during exertion (e.g., pursed-lip breathing), monitoring perceived effort with the modified Borg scale, and adopting supportive positions to manage breathlessness. The module also provides emergency and hospital contact numbers to support patients if needed.

##### 3.7.1.5 Getting started with physical activity

This module invites users to explore the perceived advantages and disadvantages of physical activity, set personalized and realistic goals using a SMART approach, and reflect on individual facilitators and barriers to engagement. It also introduces practical problem-solving strategies—such as IF/THEN planning—to help users anticipate challenges and adapt their physical activity to changing circumstances or unplanned barriers.

##### 3.7.1.6 Progression strategies

The content provides practical suggestions for gradually increasing physical activity, including step-count progression strategies.

##### 3.7.1.7 Energy conservation

To support long-term adoption of physical activity, the module also addresses practical energy conservation techniques to help manage fatigue and perform daily activities more efficiently.

##### 3.7.1.8 Action planning and coping with difficulties

This module supports users in preparing for potential setbacks, such as worsening symptoms, exacerbations, or unexpected barriers. Users are guided to develop a preventive action plan to help anticipate and manage these situations.

#### 3.7.2 B) Physical activity monitoring and progression support

A second core component of the intervention is physical activity monitoring, centered on daily step counts. The application includes an integrated step counter, allowing users to view their daily step count and track changes over time. Users can set personalized step goals, intended to support gradual progression rather than performance optimization.

#### 3.7.3 C) Motivational and supportive features

To enhance engagement and support sustained use, the application integrates several features across all components. These include motivational messages that provide positive, encouraging feedback; visual feedback features (e.g., progress bars and congratulatory messages) designed to reinforce engagement while maintaining a respectful, autonomy-supportive, and non-judgmental attitude; and reminders related to physical activity and app use, triggered by periods of inactivity. The application also provides air quality information to help users adapt outdoor activity to environmental conditions. Together, these features are intended to increase awareness of activity patterns, facilitate self-regulation aligned with self-care and Self-Determination theory, normalize fluctuations in activity, and strengthen users’ motivation to maintain physical activity despite COPD-related challenges.

#### 3.7.4 Intervention delivery

The intervention is intended to be introduced to patients at the end of their face-to-face pulmonary rehabilitation program by a trained healthcare professional. However, as the project is currently in the development phase of the MRC framework and the next step corresponds to the feasibility phase, the healthcare professional delivering the intervention will be a member of the research team (Skivington et al., 2021). To minimize potential bias outcome data will be collected by a different member of the research team who is not involved in delivering the intervention (Bhide, Shah and Acharya, 2018). In the MRC feasibility phase, the intervention delivery session will take place at the patient’s home after completion of the pulmonary rehabilitation program. This approach allows baseline measurements to be collected prior to exposure to the intervention, as the planned pilot randomized controlled trial will assess acceptability, feasibility, and preliminary effects across three measurement time points through a six-month period. Nevertheless, the intervention has been designed so that, in a future implementation phase, it could be delivered directly during the final days of the pulmonary rehabilitation program.

During the delivery session, the healthcare professional will assist the patient in installing and configuring the mobile application and will provide structured guidance on its purpose, content, and main functionalities. The intervention is designed as an asynchronous self-management digital tool, enabling participants to engage with the content independently in their home environment without real-time interaction with healthcare professionals. Participants are encouraged to use the application according to their individual needs and preferences and are invited to explore all modules and functionalities. Engagement with the full content may represent approximately 10 min per day on average, while remaining flexible in both frequency and duration of use. All content and functionalities remain continuously accessible, allowing personalized and adaptable use in daily life.

### 3.8 Expert consultation and content validation

The intervention content was validated using a two-round modified e-Delphi process with a multidisciplinary panel of nurses, pulmonologists, and physiotherapists experienced in COPD management, rehabilitation and self-management promotion. Ten experts participated in round one and seven completed round two, assessing the relevance and clarity of the intervention components. Consensus was achieved on all items in the first round, with each item-level content validity index exceeding 0.80 for both relevance and clarity, indicating strong agreement among experts. The overall calculated content validity index was excellent. No additional modifications were required in the second round, confirming the conceptual coherence, clarity, and clinical relevance of the intervention content.

## 4 Discussion

This study described the development of Respir’air BPCO, a complex self-management digital intervention designed to promote physical activity in people living with COPD following pulmonary rehabilitation. The development process was guided by the MRC framework (Skivington et al., 2021) and operationalized using the pragmatic elements proposed by Bleijenberg et al. (Bleijenberg et al., 2018), providing a structured step-by-step approach while allowing adaptation to clinical realities. This approach was essential to ensure a comprehensive and well-structured development process and to increase the likelihood of success, as it integrated evidence from a targeted literature review and umbrella review (Salgado et al., 2025, 2026), contextual analysis, input from patients and healthcare professionals, a robust theoretical underpinning, and content validation through an expert panel.

The developed intervention shares characteristics with other self-management digital interventions, such as the integration of structured educational modules, self-monitoring tools, and behavior change techniques aimed at promoting physical activity (Cox et al., 2021; Janjua et al., 2021; Liu et al., 2023; Shaw et al., 2020; Song et al., 2021; Sunjaya et al., 2022). However, Respir’air BPCO was developed using a transparent, robust, and theory-informed approach, validated by experts, that explicitly links theoretical constructs, content, behavior change techniques, and digital functionalities, thereby strengthening the clarity, coherence, and reproducibility of its mechanisms of action.

As described previously, following the MRC development phase, a feasibility phase will be conducted to assess feasibility and acceptability outcomes through a pilot randomized controlled trial. In addition, preliminary effects will be explored, including changes in physical activity levels, disease self-management, motivation, health-related quality of life, dyspnea severity, and the number of exacerbations and hospitalizations (ClinicalTrials.gov ID: NCT07262229).

It is important to acknowledge that key design decisions were shaped by the clinical and organizational context in which the project was embedded. For example, for feasibility and pragmatic reasons, the intervention was positioned as a continuation of a self-management promotion intervention to promote physical activity after pulmonary rehabilitation. This decision was supported by the observation that patients receive intensive support during inpatient rehabilitation and often achieve meaningful gains, whereas structured self-management support is limited after discharge, which may compromise the maintenance of these gains (Beauchamp et al., 2013; S. E. Desveaux et al., 2015; Jones et al., 2014b; Levack et al., 2018; Walsh et al., 2019; Watz et al., 2014). Future phases of evaluation will determine whether the intervention can be extended beyond the post-pulmonary rehabilitation context to provide structured self-management support for patients who are unable to access pulmonary rehabilitation in the first place, a group estimated to represent approximately 98% of eligible individuals according to the literature (Desveaux et al., 2015; Holland, Corso and Spruit, 2021; Spitzer et al., 2019).

A further pragmatic decision was to focus specifically on physical activity rather than addressing all components of COPD self-management, driven by the need for a feasible digital intervention with a manageable scope. However, the intervention structure was constructed to allow future expansion and integration of additional self-management components. The intervention was also designed to be asynchronous and self-paced to maximize feasibility and scalability, though this may limit direct interaction with healthcare professionals. Future developments will include synchronous sessions with healthcare professionals delivered through the platform. In addition, peer support groups and conversational agents (e.g., AI-powered chatbots) will be explored to provide continuous support, enhance user engagement, and help address constraints related to healthcare workforce availability.

A practical strength of the intervention's development was the integration of the content into an existing app infrastructure, enabling timely development and technical stability. However, this approach also introduced limitations for implementation and scalability, as integrating and maintaining content within a commercial platform involves costs related to development, licensing, updates, and long-term maintenance, and may constrain the range of functionalities that can be implemented. These factors may influence the degree of personalization, interactivity, and integration with clinical workflows and should be explicitly considered in future evaluation and implementation planning. In later stages, economic evaluation, including cost-effectiveness analysis, will be essential to inform sustainability and potential funding models. Finally, the intervention was developed in collaboration with a single pulmonary rehabilitation center, which may limit immediate transferability to other contexts, and effectiveness cannot be inferred at this stage.

From a nursing perspective, this study contributes to advancing the development of theory-informed self-management digital interventions grounded in nursing science for people living with COPD. By operationalizing the Middle-Range Theory of Self-Care of Chronic Illness within a digital format, this work illustrates how nursing theoretical knowledge can guide the design of innovative interventions that support patients’ capacity to manage chronic illness in everyday life, particularly through the promotion of physical activity. Although Self-Determination Theory is not specific to nursing science, it is highly relevant to nursing practice, as nurses frequently support motivation, and patient engagement in health behaviors. Its integration in this intervention illustrates how nursing knowledge can be enriched by complementary behavioral theories to better support sustained self-management and physical activity engagement. The intervention reflects the central role of nurses within interprofessional teams in supporting health behavior change, strengthening patient autonomy, and facilitating adaptation to chronic illness trajectories. In clinical practice, Respir’air BPCO may support continuity of care by extending self-management support beyond face-to-face pulmonary rehabilitation, a period often characterized by reduced follow-up and increased risk of physical inactivity. In nursing education, this work illustrates the relevance of integrating theoretical knowledge, self-management support approaches, and digital health perspectives to prepare nurses to support patients living with chronic conditions using innovative care modalities. In nursing research, this article provides a transparent, reproducible pathway that can support future testing, adaptation, and implementation of similar self-management digital interventions across different contexts.

## 5 Conclusion

Respir’air BPCO was developed through a structured, theory-driven, and context-sensitive process in accordance with the MRC framework for complex interventions. By integrating evidence synthesis, input from recipients and stakeholders, a robust theoretical foundation, pragmatic design decisions, and expert validation, the intervention provides a coherent self-management digital solution to promote physical activity in people living with COPD.

This work contributes to improving methodological transparency in the development of digital interventions and may inform similar initiatives in chronic disease self-management, where nurses within multidisciplinary teams have a key role in supporting effective disease self-management

## Ethics considerations

All procedures complied with the ethical principles of the Declaration of Helsinki. According to Swiss regulations, the different steps carried out during the MRC development phase did not require ethical approval.

## Declaration of generative AI and AI-assisted technologies in the manuscript preparation process

During the preparation of this work, the authors used ChatGPT to improve the clarity and style of the text of the manuscript. After using this tool, the authors reviewed and edited the content as needed and take full responsibility for the content of the published article.

## Funding statement

The MRC development phase of the intervention received no external funding.

## CRediT authorship contribution statement

**Ricardo Salgado:** Writing – review & editing, Writing – original draft, Validation, Supervision, Methodology, Conceptualization. **Patrício Costa:** Writing – review & editing, Writing – original draft, Validation, Supervision, Methodology, Conceptualization. **Philippe Delmas:** Writing – review & editing, Writing – original draft, Validation, Supervision, Methodology, Conceptualization. **José Miguel Padilha:** Writing – review & editing, Writing – original draft, Validation, Project administration, Methodology, Conceptualization.

## Declaration of competing interest

The authors declare that they have no known competing financial interests or personal relationships that could have appeared to influence the work reported in this paper.

## Data Availability

Data generated during the intervention development process are not publicly available due to ethical and data protection considerations. Aggregated and anonymized materials supporting the development process are provided in the supplementary files.
